# A Novel NAC Transcription Factor, *PbeNAC1*, of *Pyrus betulifolia* Confers Cold and Drought Tolerance via Interacting with *PbeDREBs* and Activating the Expression of Stress-Responsive Genes

**DOI:** 10.3389/fpls.2017.01049

**Published:** 2017-06-30

**Authors:** Cong Jin, Kong-Qing Li, Xiao-Yong Xu, Hu-Ping Zhang, Hui-Xian Chen, Yu-Hong Chen, Jing Hao, Yang Wang, Xiao-San Huang, Shao-Ling Zhang

**Affiliations:** ^1^Center of Pear Engineering Technology Research, Nanjing Agricultural UniversityNanjing, China; ^2^School of Life Science and Food Engineering, Huaiyin Institute of TechnologyHuaian, China; ^3^Department of Rural Development, Nanjing Agricultural UniversityNanjing, China; ^4^School of Horticulture and Plant Protection, Yangzhou UniversityYangzhou, China

**Keywords:** NAC, DREB, cold, drought, reactive oxygen species, stress-responsive genes

## Abstract

NAC (NAM, ATAF, and CUC) transcription factors are important regulator in abiotic stress and plant development. However, knowledge concerning the functions of plant NAC TFs functioning in stress tolerance and the underlying molecular basis are still limited. In this study, we report functional characterization of the NAC TF, PbeNAC1, isolated from *Pyrus betulifolia. PbeNAC1* were greatly induced by cold and drought, while salt stress had little effect on expression. PbeNAC1 was localized in the nuclei showed transactivation activity. Overexpression of *PbeNAC1* conferred enhanced tolerance to multiple stresses, including cold and drought, as supported by lower levels of reactive oxygen species, higher survival rate, higher activities of enzymes, relative to wild-type (WT). In addition, steady-state mRNA levels of 15 stress-responsive genes coding for either functional or regulatory proteins were higher levels in the transgenic plants relative to the WT with drought or cold treatment. yeast two-hybrid (Y2H) and bimolecular fluorescence complementation (BiFC) assays showed that PbeNAC1 protein can physically interact with PbeDREB1 and PbeDREB2A. Taken together, these results demonstrate that pear PbeNAC1 plays an important role in improving stress tolerance, possibly by interacting with PbeDREB1 and PbeDREB2A to enhance the mRNA levels of some stress-associated genes.

## Introduction

Plant growth and development are frequently threatened by environmental stresses such as drought and low temperature. To cope with these stresses, plants evolved a set of physiological and molecular defense mechanisms to adapt to adverse conditions (Shinozaki and Yamaguchi-Shinozaki, 2000). Over the past decades, enormous advancements have shown that, following exposure to abiotic stresses, the transcript levels of many genes undergo significant increases or decreases (Qin et al., 2011), indicating that transcriptional reprogramming plays a key role in stress adaption (Zhu, 2002; Dai et al., 2007; Huang et al., 2013).

Stress-responsive genes are generally classified into two main groups based on their products, effector molecules or regulator molecules (Huang et al., 2010). Genes in the former group encode regulatory proteins, including protein kinases, phosphatases, and transcription factors (TFs), which are responsible for transducing stress signaling and regulating expression of stress-responsive genes (Shinozaki and Yamaguchi-Shinozaki, 2000). TFs are known as master regulators that plays key roles in various biological processes, so exploitation and elucidation of stress-associated TFs and their target genes will help understand key regulons and provide valuable genes for stress tolerance improvement via genetic engineering.

NAC constitutes one of the largest TF families in plants. Proteins of this family contain a highly conserved N-terminal DNA-binding domain, which is divided into five conserved motifs, following a nuclear localization signal sequence and a variable C-terminal domain (Riechmann et al., 2000). Although more than 100 members of this family are widely distributed in the plant genome, only a few of them have been reported NAC TFs have been shown to function in the transcriptional regulation of a diversity of biological processes, including lateral root formation (Xie et al., 2000; Hao et al., 2011), shoot branching (Mao et al., 2007), adventitious shoot formation (Hibara et al., 2003), leaf senescence (Balazadeh et al., 2011), flavonoid biosynthesis (Morishita et al., 2009), embryogenesis (Larsson et al., 2011), seed development (Verza et al., 2011), cell division (Kim et al., 2006) and cell wall development (Zhao et al., 2010). More recently, some plant NAC TFs were found to be involved in plant responses to biotic and abiotic stresses (Mao et al., 2012). For example, overexpression of *ATAF1* and *ATAF2* increased susceptibility to fungal pathogens in *Arabidopsis* (Wu et al., 2009), transgenic rice plants expressing *OsNAC6* enhanced dehydration, high salinity and disease tolerances (Nakashima et al., 2007), *StNAC* gene was found to be involved in the response to pathogen attack and wounding in potato (Collinge and Boller, 2001), *BnNAC* genes from *Brassica* were induced by drought and cold (Hegedus et al., 2003), *SNAC1/2* improved drought resistance and salt tolerance in rice (Hu et al., 2006, 2008; Kaneda et al., 2009), and in *Arabidopsis* transgenic plants, *ANAC019*, *ANAC055*, and *ANAC072* overexpression increased drought stress tolerance (Tran et al., 2004). AtNAC2, a TF downstream of ethylene and auxin signaling pathways, is involved in salt stress response and lateral root development (He et al., 2005).

During the past decades, enormous progress has been made in deciphering significant components implicated in the cold signaling network (Thomashow, 2001; Chinnusamy et al., 2006; Huang et al., 2013; Peng et al., 2014; Wisniewski et al., 2014). One of the most important milestones has been the identification of C-repeatbinding factor (*CBF*) genes, including CBF1/DREB1B, CBF2/DREB1C, and CBF3/DREB1A (Yamaguchi-Shinozaki and Shinozaki, 2005; Medina et al., 2011). The *CBF*s, conserved among monocots and dicots, play crucial roles in regulating a large spectrum of cold-regulated (COR) genes, collectively called the CBF regulon, implying that they act as central hub in the cold signaling network (Nakashima et al., 2009; Thomashow, 2010; Wisniewski et al., 2014). Previous studies related to CBFs were mainly characterized in the model plants, while knowledge of the molecular mechanisms underlying enhanced abiotic stress tolerance of CBFs remain poorly understood, especially in the woody plants. Recently, roles of CBFs in non-model plants are being gradually revealed, such as, PtrBAM1 isolated from *Poncirus trifoliata* is a member of CBF regulon and plays an important role in cold tolerance by modulating the levels of soluble sugars acting as osmolytes or antioxidants (Peng et al., 2014). In another work, they showed that that CBF1 functions in cold tolerance via regulating CORc115 expression in Citrus (Champ et al., 2007; Wisniewski et al., 2011). These findings suggest that CBF acts as a master regulator of the cold signaling pathway and plays a pivotal role in mediating plant responses to cold, thus establishing CBF-COR as the most significant signaling cascade.

More recently, some interesting studies showed that some NAC TFs were involved in *DREB/CBF-COR* pathway. For example, GmNAC20 regulates COR genes and mediate stress tolerance by binding directly to the promoter of DREB1A and suppressing the expression of DREB1C (Hao et al., 2011). AtVIN2 directly binds to the promoters of *COR* and *RD* genes to regulate them in *Arabidopsis* (Yang et al., 2011). Shan et al. (2014) reported that MaNAC1 is involved in cold stress through interacting with MaCBF1 in Banana. These findings indicated that NACs acts as a pivotal role in mediating plant responses to abiotic stress, thus establishing NAC-DREB/CBF-COR as the significant signaling cascade, which enable us to further exploring this regulatory mechanism.

*Pyrus betulifolia* originated in Northern China, which is extremely cold-hardy and drought resistance (Li et al., 2016), making it an ideal source from which to isolate genes of agronomical importance with potential use for genetic engineering. In this study, we report that overexpression of *P. betulifolia NAC1* (designated as *PbeNAC1*) led to enhanced cold and drought tolerance in transgenic lines. Both yeast two-hybrid and BiFC assays revealed that the PbeNAC1 was associated with PbeDREB1/CBF and PbeDREB2A/AP2, and also enhanced the transcript levels of some stress-responsive genes. Taken together, our data demonstrate that *PbeNAC1* is involved in the regulation of cold and drought tolerance via modulating the expression of stress-responsive genes through interacting with the PbeDREB1 and PbeDREB2A.

## Materials and Methods

### Plant Materials and Stress Treatments

Fourty-five-day-old *P. betulifolia* plants grown in the seedling beds at Center of Pear Engineering Technology Research, Nanjing Agricultural University, were used to gene cloning and expression analysis of *PbeNAC1* under different treatment. Uniform and healthy seedlings were inserted in a flask containing distilled water, which were kept for 1 day in a growth chamber at 25°C with 16-h light/8-h dark photoperiod (45 μmol m^-2^ s^-1^) being treated with various treatments. For cold stress, the seedlings were placed in the growth chamber set at 4°C for 72 h; the leaves were collected at 0, 5, 12, 24, 48, and 72 h after initiation of the treatment. For dehydration treatment, the seedlings were placed on dry filter papers above a work bench and dried at 25°C (in autumn, with relative humidity of 44.0%) for 0, 0.5, 1, 3, and 6 h. For the salt treatment, the seedlings were incubated in the 200 mM NaCl solution for 0, 1, 5, 12, 24, and 48 h. ABA treatment was applied by immersing the seedlings in 100 μM ABA solution for 0, 1, 6, 12, 24, and 48 h. In order to eliminate the effects of hypoxia or anoxia creased, we set up another experiments of water as the control corresponding to ABA or salt treatment. For each treatment, a minimum of 60 seedlings were used, and the sampled were then frozen immediately in liquid nitrogen and stored at -80°C super low temperature freezer until use for gene expression analysis.

### RNA Extraction and qPCR Analysis

Total RNA was extracted from the leaves using TRIZOL reagent (Invitrogen, United States). Approximately 1 μg of total RNA was reversely transcribed into cDNA using the PrimeScript^®^ RT reagent kit (Takara, China) according to the manufacturer’s instructions. Quantitative real-time PCR was carried out with SYBR-Green PCR kit (TakaRa) using 20 μL of reaction mixture consisting 10 μL of 2 × SYBR-PreMix EX Taq, 100 ng of cDNA, and 0.25 μM of each primer (GSP1, Supplementary Table S1). The program of the qPCR was as follows: denaturation at 94°C for 5 min, followed by 40 cycles of 94°C for 10 s, 60°C for 30 s, 72°C for 30 s, and a final extension 3 min at 72°C. Each sample was analyzed in four replicates, and each sample was amplified in four independent replicates. Relative gene expression was calculated according to the 2^-ΔΔCt^ method (Livak and Schmittgen, 2001) of the system. *Tubulin* and *Ubiquitin* were analyzed in parallel as reference controls for *P. betulifolia* and tobacco, respectively, to normalize expression levels.

### Gene Isolation and Bioinformatics Analysis

A pear sequence with a full-length, highly homologous to NAC1, was obtained by sequence search against the pear genome database^1^. Based on the above sequence, a pair of gene-specific primers (GSP2; Supplementary Table S1) was used for RT-PCR to amplify *PbeNAC1*. RNA extraction and cDNA synthesis of the relevant samples were carried out as elaborated above. After treatment with RNase-free DNase I, 1 μg of total RNA was used to synthesize first-strand cDNA with the RevertAid^TM^ First Strand Synthesis Kit (TOYOBO, Japan). PCR was performed in a total volume of 50 μL containing 300 ng cDNA, 10 μL of 1 × TransStart FastPfu buffer, 5 μL of 2.5 mM deoxyribonucleotide (dNTP), 0.4 μM of a pair of specific primers (GSP2, Supplementary Table S1), 2.5 units of TransStart FastPfu DNA polymerase and nuclease-free water. PCR was performed in a thermocycler with a program consisting of 2 min at 95°C, followed by 40 cycles of 20 s at 95°C, 20 s at 55°C, 60 s at 72°C, and a 5 min extension at 72°C. The PCR product was purified, subcloned into pMD18-T vector (Takara, China) and sequenced in Invitrogen (Shanghai, China). The multiple alignments of the deduced amino acid sequence were performed using the ClustalW program. A phylogenetic relationship tree was constructed by the Maximum Likelihood (ML) method using MEGA (version 6.0). Theoretical isoelectric point (*p*I) and molecular weight were also identified by tools in an internet server, ExPASy (Expert Protein Analysis System^2^).

### Transactivational Activity and Subcellular Localization of *PbeNAC1*

The full-length ORF of PbeNAC1 without a stop codon was amplified by RT-PCR with primer pairs (GSP3; Supplementary Table S1), and the product was cloned into pMN6 vector to test its transcriptional activation activity. The fusion plasmid PbeNAC1-pMN6 together with an internal control vector pRNL and a reporter plasmid pGLL were co-transformed into *Arabidopsis* mesophyll protoplasts by PEG method (Lee et al., 2003). After incubation at 22°C for 24 h, the protoplasts were collected to assay the signals of Firefly and Renilla luciferase using the dual luciferase assay reagents (Promega) according to the manufacturer’s protocol.

The whole ORF of the *PbeNAC1* gene was amplified by RT-PCR using primers (GSP4; Supplementary Table S1) containing *Bgl*II and *Spe*I restriction sites, The PCR products were digested by the same restriction sites and cloned into pCAMBIA1302 vector to generate a fusion construct (p35S-PbeNAC1-GFP). After confirmation by sequencing, the fusion construct (p35S-PbeNAC1-GFP) and the control vector (pCAMBIA1302) was transferred into *Agrobacterium tumefaciens* strain GV3101 using the freeze-thaw method. Transient transformation of tobacco leaves was done as described by Yang et al. (2000). The transformed leaves were then cultured on MS medium for 48 h, followed by live cell imaging under an inverted confocal microscopy (Zeiss LSM 780, Germany).

### Transformation and Characterization of Transgenic Plants

The pMD-18T-*PbeNAC1* was restricted with *Bgl*II and *BstE*II, and the resulting product was cloned into *Bgl*II and *BstE*II sites of the binary vector pCAMBIA1301 to generate pCAMBIA1301-*PbeNAC1*, which was introduced into *A. tumefaciens* strain GV3101 by the freeze-thaw method. About 50-days-old tobacco seedlings were used for transformation based on a leaf disk transformation method (Horsch et al., 1985). Transgenic plants were cultured on selection medium containing hygromycin as a selective marker. Hygromycin resistant seedlings were transferred to rooting medium. Putative transgenic T_0_ plants were maintained in a growth chamber at 25°C with a 16 h light/8 h dark photoperiod until they flowered and set seeds. Homozygous lines were harvested according to an earlier method (Huang et al., 2015a). After obtaining the transgenic tobacco seedlings, the genomic DNA was extracted from *in vitro* T_0_ plants and the WT using cetyltrimethyl ammonium bromide (CTAB). To verify the transgenic tobacco plants, the 25 μL PCR reaction conditions contained 2.5 μL10 × PCR buffer, 2 μL of 2.5 mM dNTPs, 0.25 μM of each primer, and 2.5 units of Taq polymerase. The PCR reaction conditions were performed at 94°C for 3 min, followed by 35 cycles of 30 s at 94°C, 30 s at 60°C, 1 min 30 s at 72°C, and a final extension at 72°C for 10 min. PCR products were electrophoresed on 1.2 % (w/v) agarose gel containing 0.5 μg/L ethidium bromide and visualized under UV transillumination. Overexpression of *PbeNAC1* was analyzed by semi-quantitative RT-PCR according to Huang et al. (2010) with a minor modification. Homozygous tobacco plants at the T_2_ generation plants were used for a stress tolerance assay and physiological measurement.

Seeds of T_2_ generation transgenic plants and wild type were sterilized for 30 s in 70% (v/v) ethanol and incubated in 2.5% (v/v) H_2_O_2_ for 5 min, followed by rinse with sterile distilled water for three times before they were sown on germination medium (GM) containing MS salts, 30 g L^-1^ sucrose and 0.8% agar (pH 5.8). For freezing treatment, the seedlings were first transplanted into plastic containers filled with a mixture of soil and sand (1:2) in the same chamber with a 16 h photoperiod and 40% relative humidity. 30-days or 60-days-old tobacco plants from WT and transgenic lines grown under normal conditions were directly treated for 24 h at 0°C without cold acclimation, and then moved to ambient environment for further growth. Survival rate was scored after 1 day recovery growth; photos were taken before and after the freezing treatment and after the recovery.

Dehydration stress was performed as described by Gong et al. (2015) with slight modification. The fresh weight (FW) of the seedlings was measured every 15 min using a scale, and the percentage FW loss was determined relative to the initial weight. For drought experiment, 15-days-old transgenic tobacco lines and WT were first transplanted into plastic pots (10 cm diameter) filled with a mixture of soil and sand (1:1) where they were regularly watered for 30-days-old or 60-days-old in a growth chamber (25 ± 2°C, relative humidity 65%, photoperiod 16 h light/8 h dark with a light intensity of 45 μmol m^-2^s^-1^) (unless otherwise stated, all chamber conditions are the same) until the drought treatment. The plants were deprived of watering for 15 days, and then returned to regular irrigation for 5 days, and then the leaves were collected for analysis of antioxidant enzyme activity, cell death, relative water content (RWC), metabolite levels, and expression of stress-responsive genes.

### Physiological Measurement and Histochemical Staining

Electrolyte leakage (EL), MDA, and total chlorophyll content were measured according to previous studies (Huang et al., 2010; Wang et al., 2011). Proline content was assessed as described by Zhao et al. (2009) with slight modification. Cell death was examined with trypan blue staining based on the method of Pogány et al. (2004). Antioxidant enzyme (POD and CAT) activity, H_2_O_2_ level, and anti-superoxide anion activity were quantified using the relevant detection kits (Nanjing Jiancheng Bioengineering Institute) based on the manufacturer’s instructions. Histochemical staining with DAB and NBT was used to analyze the *in situ* accumulation of H_2_O_2_ and O_2_^-^ according to Huang et al. (2011).

### Y2H and BiFC Assays

To elucidate the molecular mechanisms underlying the enhanced stress tolerance of transgenic plants overexpressing *PbeNAC1*, the full-length cDNA of PbeDREB1 and PbeDREB2A were amplified using primer pairs GSP6 and GSP7 (Supplementary Table S1), respectively, and cloned into pGBKT7 vectors to get BD-PbeDREB1 and BD- PbeDREB2A, while the full-length PbeNAC1 was amplified with primer (GSP5; Supplementary Table S1), and inserted into pGADT7 (clontech). The two constructs were co-transformed into yeast cells AH109, which were cultured on SD/-His/-Leu/-Trp plates with or without 3-AT to identify the DNA-protein interaction.

For bimolecular fluorescence complementation (BiFC) analysis, the PbeDREB1 and PbeDREB2A ORF without a stop codon were PCR amplified with primer pairs GSP6 and GSP9, respectively, and subcloned into pUC-pSPYNE-35S containing the N-terminal fragment of yellow fluorescent protein (nYFP) to get PbeDREB1-nYFP and PbeDREB2A-nYFP. Meanwhile, the full-length cDNA of PbeNAC1 without a stop codon was amplified using primer pair GSP8 and inserted into pUC-pSPYNE-35S containing the C-terminal fragment of YFP (cYFP) to generate PbeNAC1-cYFP. Transient expression assay of PbeNAC1 in *Arabidopsis* was carried out by Zhao et al. (2013). YFP fluorescence in was observed via a confocal laser scanning microscope (LSM510 Meta, Zeiss, Germany).

### Statistical Analysis

Each stress treatment was repeated at least three times with consistent results. Data are presented as means ± SE of at least three independent replicates from one representative experiment. The data were analyzed by Duncan’s multiple range tests in the ANOVA program of SPSS (IBM SPSS 22), taking ^∗^*P* < 0.05, ^∗∗^*P* < 0.01, ^∗∗∗^*P* < 0.001 as significantly different.

## Results

### *PbeNAC1* Cloning and Sequence Analysis

**FIGURE 1:**
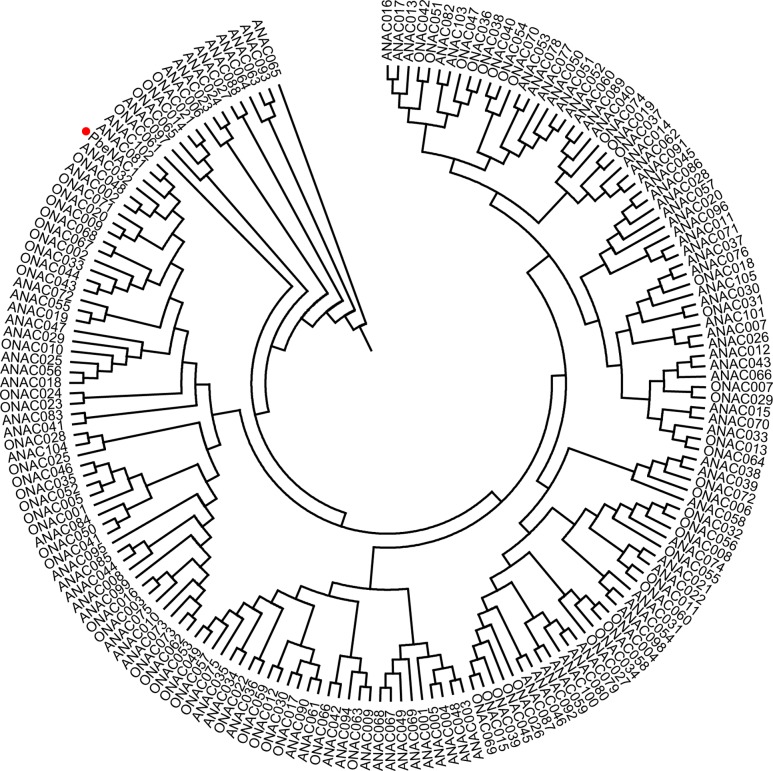
A Phylogenetic tree analysis of PbeNAC1 and NACs amino acid sequences of *Arabidopsis thaliana* and rice using MEGA 4.0. The Phylogenetic tree was tested using the bootstrap method with 1,000 replicates by the Neighbor–Joining (NJ) method.

**FIGURE 2:**
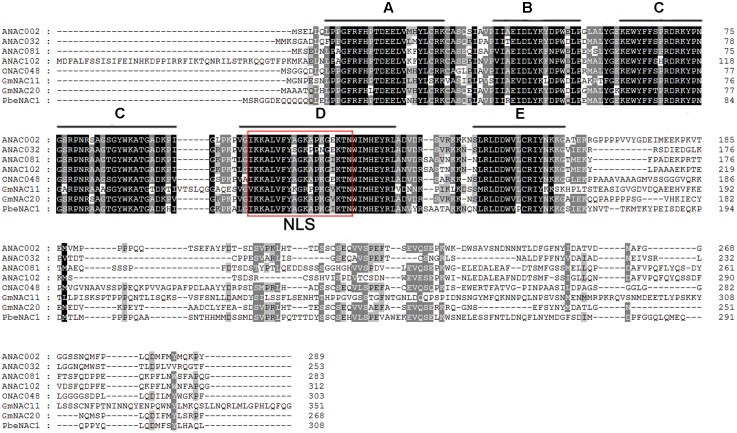
Multiple alignments of PbeNAC1 protein and other plant NAC proteins from *A. thaliana* (ANAC002, At1G01720; ANAC032, At1G77450; ANAC081, At5G08790 and ANAC102, At5G63790); *Oryza sativa* (ONAC048, AK068392); and Glycine max (GmNAC11, EU44035, and GmNAC20, EU440353). The five highly conserved subdomains (**A–E**) and the nuclear localization signal (NLS) are shown by black lines and red box, respectively.

To get the *PbeNAC1* gene, we first searched the pear genome database using the OsNAC1 sequence as a query, which yielded different outputs. Homology comparison in the NCBI database showed that the first output exhibited high sequence homology to *NAC1*. So, based on this output sequence a pair of primers was designed for RT-PCR to amplify the ORF of NAC1 gene in *P. betulifolia*. Analysis in ORF Finder indicates that it contained a complete ORF of 927 bp, flanked by a 246 bp 5′-untranslated region (UTR) and a 139 bp 3′-UTR. The cDNA, designated as *PbeNAC1*, contains an intact ORF and encodes a polypeptide of 308 amino acids with a calculated molecular mass of 35.6 kDa and an isoelectric point of 6.46. The gene has been deposited in NCBI and the accession number of KU663372. A phylogenetic tree constructed based on the sequences of PbeNAC1 and NACs of other plants, such as *Arabidopsis thaliana*, and rice. The phylogenetic tree indicated that PbeNAC1 was closely related to ANAC081 (**Figure 1**). Multiple sequence alignments indicated that PbeNAC1 shares a significant degree of sequence identity with seven other NAC proteins. The alignment showed that PbeNAC1 has a highly conserved sequence in their N-terminal regions, including five conserved subdomains (A–E), and PbeNAC1 possessed a conserved nuclear localization signal (NLS, positions 114–132 aa) (**Figure 2**).

### Expression Patterns of *PbeNAC1* under Various Treatments

**FIGURE 3:**
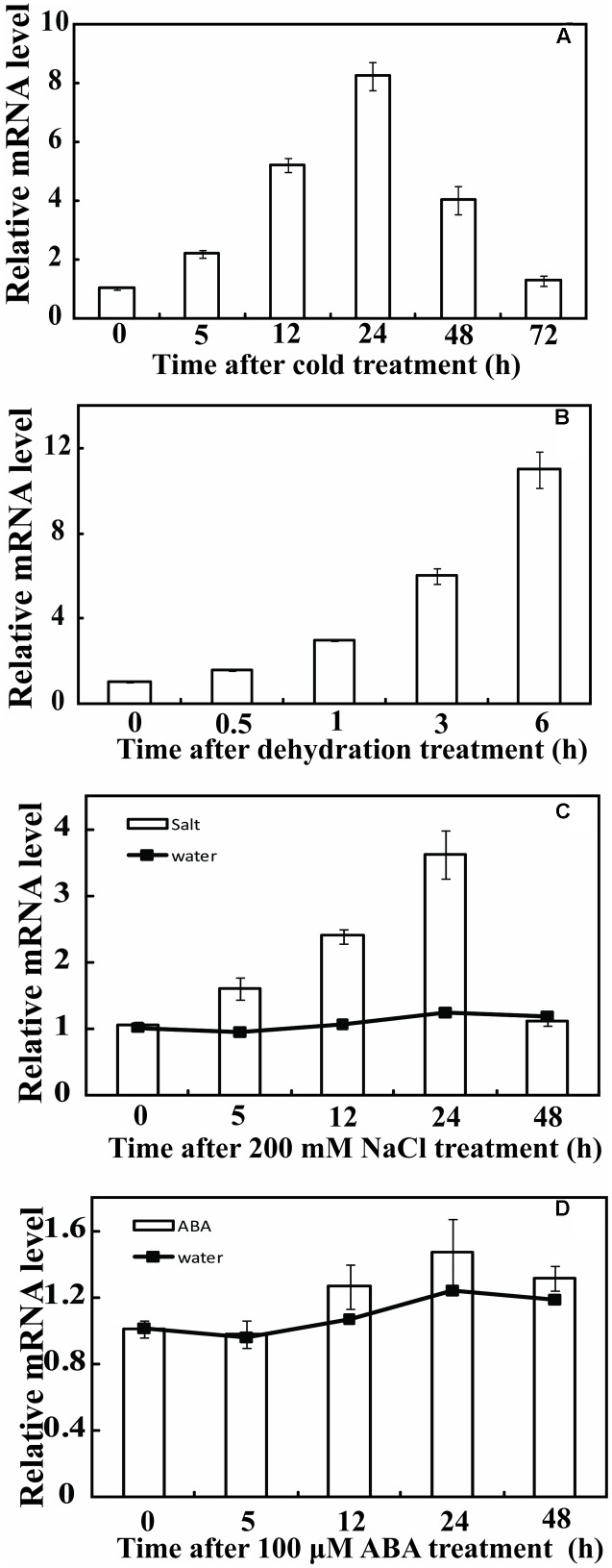
Relative expression pattern of *PbeNAC1* in *Pyrus betulifolia* under different stress treatments. Expression profiles of *PbeNAC1* was investigated by qRT-PCR using *P. betulifolia* subjected to various stress treatments, such as 4°C **(A)**, dehydration **(B)**, 200 mM NaCl **(C)** and 100 μM ABA **(D)**.

Real-time Quantitative PCR (qPCR) was used to analyze the expression profiles of *PbeNAC1* under various abiotic stresses, including cold (4°C), dehydration, salt and ABA. Under normal growth conditions, transcript levels of *PbeNAC1* underwent minor changes (data not shown). By contrast, *PbeNAC1* was gradually induced within 1 day of cold treatment, but was sharply up-regulated at 2 days to nearly eightfold of its initial level and declined at the last day (**Figure 3A**). When the seedlings were treated with dehydration, *PbeNAC1* mRNA abundance was slightly induced at 0.5 h, followed by progressive elevation until reaching the peak value at 6 h, which was an approximately 12-fold increase relative to the initial level (**Figure 3B**). We also examined the transcript levels to test whether *PbeNAC1* is responsive to salt. As shown in **Figure 3C**, treatment with 200 mM NaCl led to a quick accumulation of *PbeNAC1* mRNA level, which progressed until a maximum level was reached at 2 days. However, treatment with ABA did not change notably transcript level of *PbeNAC1* except a slight decrease at 1 day, indicating that *PbeNAC1* was not ABA-inducible (**Figure 3D**). The results also showed that the effect of water occurred hypoxia or anoxia on plant was negligible during the 48 h (**Figures 3C,D**).

### PbeNAC1 Was Localized in the Nucleus with Transactivation Activity

**FIGURE 4:**
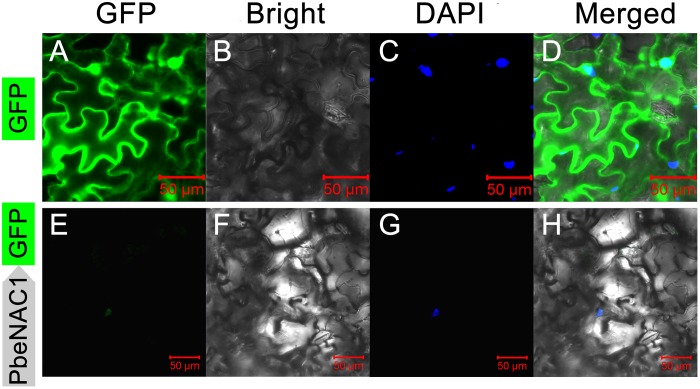
Subcellular localization of PbeNAC1 in *Nicotiana benthamiana* leaves. The control vector (GFP alone, **A–D**) and fusion construct (PbeNAC1-GFP, **E–H**) were separately transiently expressed in 5-week-old *N. benthamiana* leaves by agroinfiltration and all images were collected under the Zeiss confocal microscope after agroinfiltration for 48 h. DAPI images indicate nuclear staining.

Sequence analysis showed that PbeNAC1 contained a nuclear localization signal (aa 114–132), implying that it may be localized in the nucleus. To confirm this, the PbeNAC1 coding region was fused to the N-terminus of the GFP reporter gene under the control of the cauliflower mosaic virus 35S promoter. Microscopic observation showed that the control GFP was uniformly distributed throughout the whole cell (**Figures 4A–D**), while the PbeNAC1–GFP fusion protein was observed exclusively in the nucleus (**Figures 4E–H**), indicating that PbeNAC1 was a nuclear protein.

**FIGURE 5:**
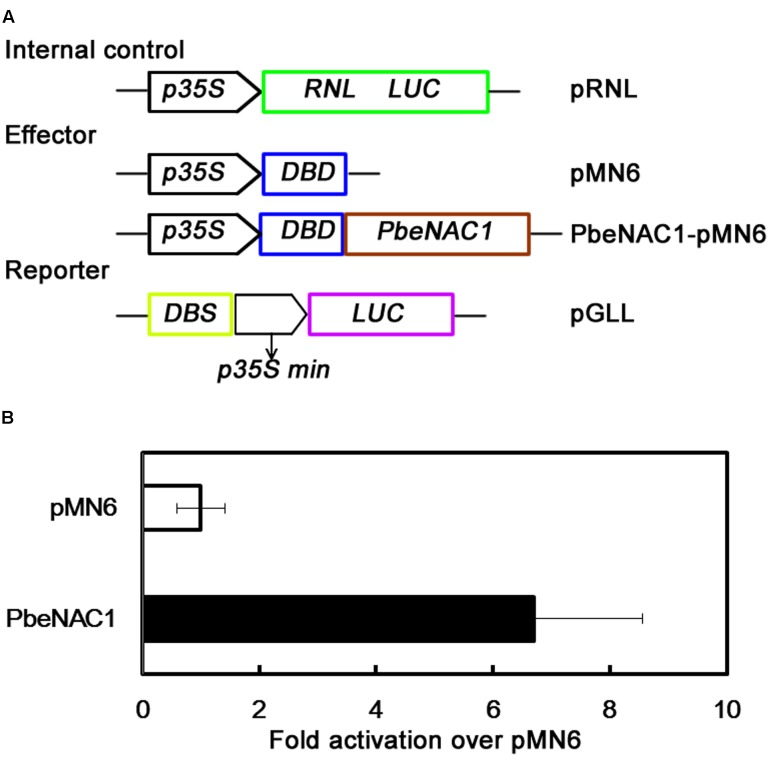
Analysis of transcriptional activation of PbeNAC1 in *Arabidopsis* protoplasts. **(A)** Construction of schematic diagram used for PbeNAC1 transcriptional activation assay in the pMN6 system. LUC, firefly luciferase; REN LUC, Renilla luciferase. **(B)** The ratio between the transcriptional activation of PbeNAC1 and pMN6 in *Arabidopsis* protoplasts. pMN6 represents vector-transfected cells, PbeNAC1 represent PbeNAC1-expressing cells.

To further identify if PbeNAC1 functions as a transcriptional activator, the transient expression assay system was used. For this purpose, the full-length PbeNAC1 coding region was fused to pMN6 vector to generate a fusion construct PbeNAC1-pMN6 under the control of the 35S promoter. The report construct contained a *Luciferase* (*LUC*) reporter gene driven by a minimal *35S* promoter with the GAL4 DNA binding site (DBS) (**Figure 5A**). The *Renilla Luciferase* (*RNL LUC*) gene under a control of a 35S promoter served as an internal control. These constructs were expressed transiently in protoplast cells from 5-week-old *Arabidopsis* plants under a 16-h-dark/8-h-light photoperiod. Expression of *PbeNAC1* in the system significantly stimulated *LUC* expression, thus more effective activated *LUC* reporter gene activity, of the control was less than sixfolds of the those in the presence of *PbeNAC1* (**Figure 5B**). These data exhibited that PbeNAC1 had transactivation activity.

### Enhanced Cold Tolerance in the Transgenic Tobacco

Given *PbeNAC1* was induced by cold and dehydration in a stronger manner, we speculated that *PbeNAC1* might play a key role in the regulation of cold and drought stress response. To examine this hypothesis, we first generated transgenic tobacco plants overexpressing *PbeNAC1* via *Agrobacterium*-mediated transformation. Two independent overexpression lines of tobacco (OE3 and OE7) with high transcript levels of *PbeNAC1* were used for cold and drought tolerance analysis (Supplementary Figure S1).

**FIGURE 6:**
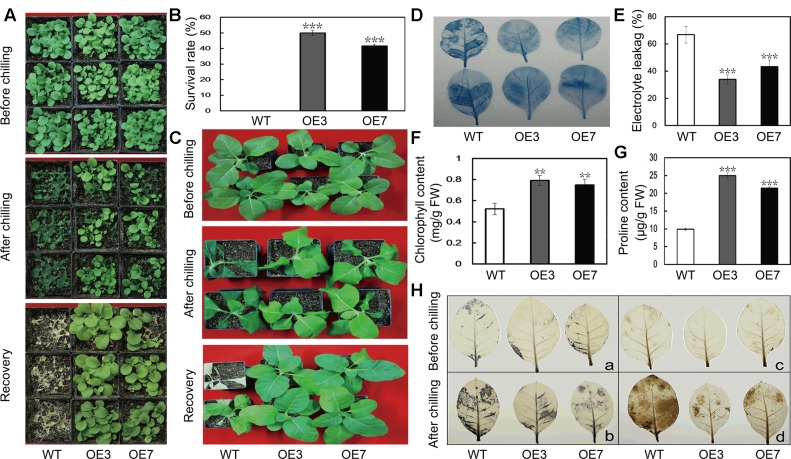
Cold tolerance analysis of transgenic tobacco plants overexpressing *PbeNAC1*. **(A)** Plant phenotype of 30-days-old transgenic tobacco (OE3 and OE7) and WT before and after freezing treatment (0°C) for 24 h, followed by recovery growth for 10 days in an ambient environment. **(B)** Survival rates of PbeNAC1 overexpression lines and WT after 10 days of recovery from freezing treatment. **(C)** Photographs of 60-days-old WT and transgenic plants before and after freezing treatment, followed by recovery at room temperature for 10 days. **(D–G)** Cell death **(D)**, Electrolyte leakage (EL) **(E)**, chlorophyll content **(F)** and Proline content **(G)** in the transgenic lines and WT measured after freezing treatment. **(H)** Accumulation of O_2_^-^ and H_2_O_2_ in the WT and transgenic lines (OE3 and OE7) under freezing treatment, as monitored with NBT (a,b) and DAB (c,d), respectively. Asterisks indicate that the value is significantly different from that of the WT at the same time point (^∗∗^*P* < 0.01; ^∗∗∗^*P* < 0.001).

To evaluate the function of *PbeNAC1* in cold tolerance, T_2_ transgenic tobacco plants and the WT were subjected to cold treatment at 0°C. Under normal growth conditions, the transgenic plants were morphologically indistinguishable from the WT. When 30-day-old seedlings were treated for 24 h at 0°C, the WT plants suffered more serious freezing injuries compared with the transgenic lines (**Figure 6A**). After recovery at room temperature for 10 days, the survival rate of WT plants was 0, significantly lower than that of the transgenic lines: 48.3% for OE3 and 41.6% for OE7 (**Figure 6B**). When 60-days-old transgenic seedlings and WT tobacco plants were exposed to freezing stress, the leaves of the WT plants showed severe freezing injury, whereas the two transgenic lines were affected to a less extent (**Figure 6C**). Cell death is a reliable indicator of cell damage caused by abiotic stresses. We can observe the comparably extent of staining by trypan blue in the leaves between the WT and the two transgenic lines without freezing stress, whereas deeper staining occurred in the leaves of the WT compared with the transgenic plants after the freezing stress for 20 h, demonstrating that more serious cell death occurred in the WT (**Figure 6D**). In another experiment, 60-day-old plants were exposed to 20 h freezing stress. In order to compare the physiological differences, EL, chlorophyll and Pro content were measured in the samples collected after 20 h of freezing stress. EL of OE3 (33.9%) and OE7 (43.2%) was significantly lower than the 66.8% found in the WT (**Figure 6E**). While total chlorophyll contents in the two transgenic lines were about 1.5 times higher than that of WT (**Figure 6F**). We also measured proline contents in the transgenic lines and WT after freezing treatment, as this compound has been considered as an important metabolite indicating the relevance to stress tolerance. As shown in **Figure 6G**, the transgenic lines accumulated higher proline in comparison with the WT. These results revealed that transgenic overexpression of *PbeNAC1* improved freezing tolerance in tobacco plants.

### Overexpression of *PbeNAC1* Improves Tolerance to Drought Stress

**FIGURE 7:**
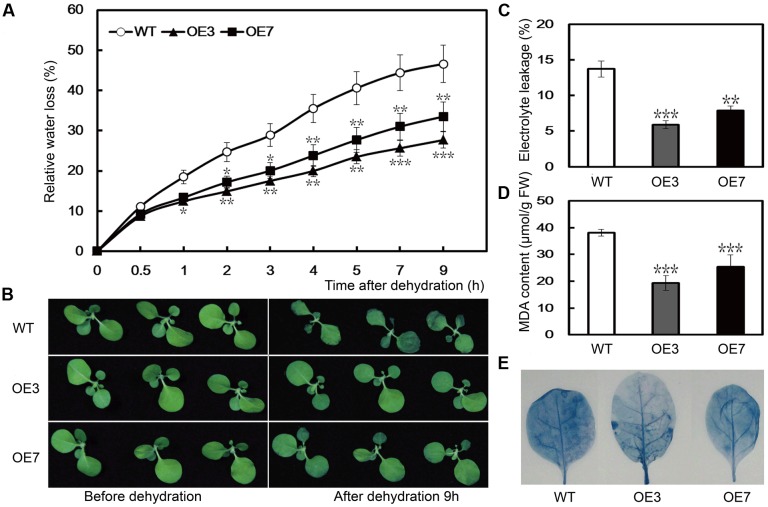
Dehydration stress resistance analysis of transgenic lines. **(A)** Phenotypes of 30-days-old WT tobacco and the two transgenic lines (OE3 and OE7) before (left) and after (right) dehydration for 9 h at ambient environment. **(B)** Fresh water loss rate of WT, OE3, and OE7 during a 9 h dehydration stress. **(C–E)** EL **(C)**, MDA **(D)**, and cell death **(E)** in the transgenic lines and WT determined after dehydration stress. Asterisks indicate that the value is significantly different from that of the WT at the same time point (^∗∗^*P* < 0.01; ^∗∗∗^*P* < 0.001).

To evaluate the function of *PbeNAC1* in drought tolerance, we performed a dehydration tolerance assay using T_2_ plants of the two lines (OE3 and OE7) as this gene exhibited a quick response to dehydration. When the 30-days-old *in vitro* seedlings were dehydrated in an ambient environment, a steady decrease in FW was observed in both the WT and the two transgenic lines. However, after 9 h of dehydration, the water loss rate in the WT was 46.6%, significantly higher than that of OE3 (27.7%), and OE7 (32.5%) (**Figure 7A**). Although OE7 lost less water than OE3, the difference was not statistically significant after dehydration. At the end of dehydration, phenotype differences were prominent between the WT and transgenic lines, as the former lost its turgor to a greater extent (**Figure 7B**). As EL is an indicator of membrane injury, the EL was compared between the WT and the transgenic plants after 9 h of dehydration. As shown in **Figure 7C**, the EL was significantly higher in the WT (49.7%) than in OE3 (30.8%) or OE7 (9.4%), indicating that the WT suffered from severe membrane damage. MDA, an important lipid oxidation product, can reflect the extent of damage of the membranes. The assay indicated that the MDA contents of OE3 (5.9 μmol/g FW) and OE7 (7.9 μmol/g FW) were significantly lower than the WT (13.7 μmol/g FW; **Figure 7D**). In addition, slighter cell death staining was visualized in the two transgenic lines after dehydration for 9 h. These findings demonstrated that overexpression of *PbeNAC1* could alleviate the membrane damage in the transgenic plants (**Figure 7E**). These results showed that overexpression of *PbeNAC1* led to an elevation of the dehydration tolerance in the transgenic tobacco.

**FIGURE 8:**
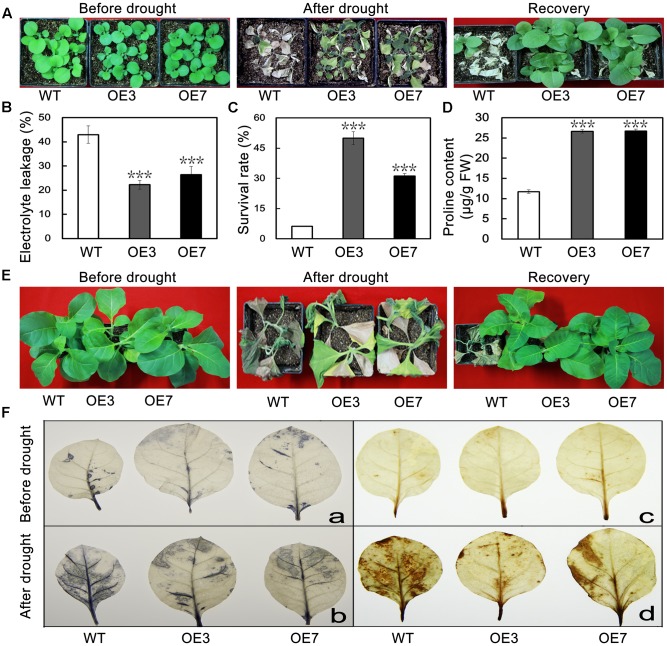
Overexpression of *PbeNAC1* confers enhanced drought resistance in transgenic lines. **(A)** Representative photographs of 30-days-old WT and the two transgenic lines (OE3 and OE7) drought for 15 days, followed by recovery for 7 days. **(B)** Phenotypes of 60-days-old WT and the two transgenic lines before and after drought stress for 15 days, After normal growth for 7 days. **(C–E)** EL **(C)** survival rate **(D)** and proline content **(E)** in the WT and transgenic lines measured after drought stress for 15 days. **(F)** Detection of H_2_O_2_ and O_2_^-^ accumulate in leaves of transgenic and WT plants normal and unnormal condition based on histochemical staining with NBT (a,b) and DAB (c,d), respectively. Asterisks indicate that the value is significantly different from that of the WT at the same time point (^∗∗∗^*P* < 0.001).

To evaluate the effect of *PbeNAC1* overexpression lines on drought tolerance, when 30-days-old tobacco plants were deprived of water for 15 days, followed by re-watering for 7 days. After 15 days without water, leaf wilting was observed in all tested plants; obviously the two transgenic lines showed better leaf turgor compared to the WT. After watering was resumed, the transgenic plants recovered their growth more rapidly than did the WT plants. The survival rates of the transgenic lines were significantly higher than that of WT (**Figure 8A**). When 60-days-old tobacco plants were used for the drought treatment, similar results were obtained: leaf wilting was more evident in the WT relative to the two transgenic lines (**Figure 8B**). Consistent with the enhanced drought tolerance phenotype, lower levels of EL, higher survival rates, and higher Pro content (**Figures 8C–E**). These results suggest that overexpression of *PbeNAC1* had a significant effect on the improvement of drought resistance in the transgenic tobacco plants.

### ROS Levels and Antioxidant Enzyme Activities

**FIGURE 9:**
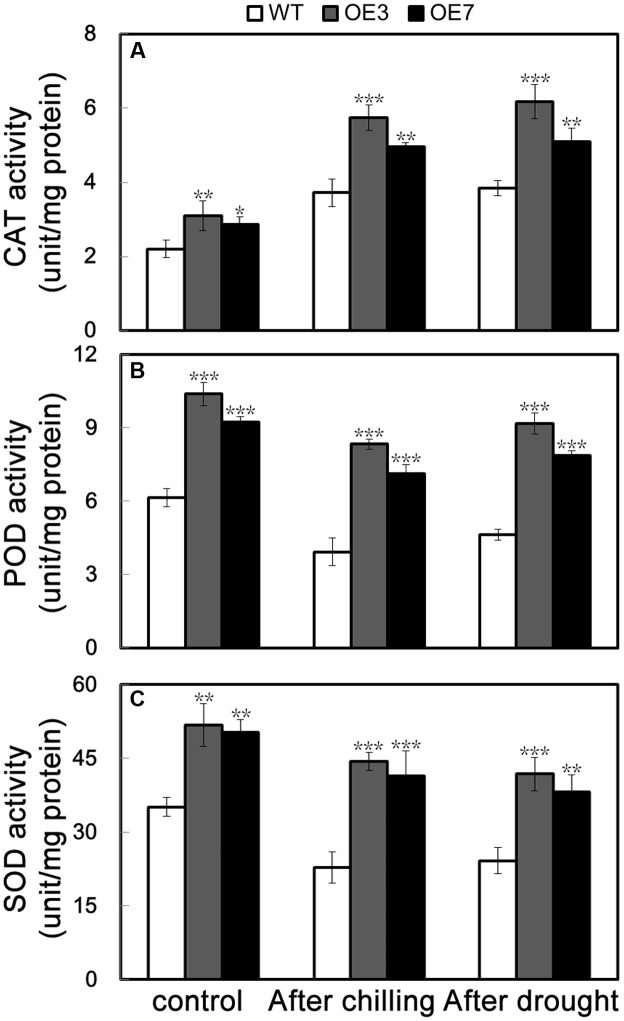
Activities of antioxidant enzymes in WT, transgenic lines (OE3 and OE7), WT. **(A–C)** Activities of CAT **(A)**, POD **(B)**, and SOD **(C)** of WT, OE3, and OE7 plants under normal conditions and under cold and drought stress. Asterisks indicate that the value is significantly different from that of the WT at the same time point (^∗^*P* < 0.05; ^∗∗^*P* < 0.01; ^∗∗∗^*P* < 0.001).

The above data suggest that the transgenic plants overexpressing *PbeNAC1* exhibited enhanced tolerance to cold, dehydration and drought. It is well documented that abiotic stresses usually result in elevation of cellular ROS level; thus the ROS level is an indicator of stress tolerance. Histochemical staining by DAB and NBT was used to reveal *in situ* accumulation of H_2_O_2_ and O_2_^-^, respectively. After freezing stresses for 20 h, the WT leaves displayed deeper staining patterns when compared with OE3 and OE7, in line with the quantitative measurement of ROS (**Figure 6H**). In addition, the ROS level in the leaves sampled from the 60-days-old potted plants exposed to drought for 10 days was also checked. Similarly, OE3 and OE7 exhibited clearly less intense DAB and NBT staining in comparison with the WT (**Figure 8F**). Taken together, these data suggest that the transgenic plants accumulated lower levels of H_2_O_2_ and O_2_^-^ under freezing and drought stresses. The above-mentioned results demonstrated that the two transgenic lines contained fewer ROS relative to WT. Since enzymatic and non-enzymatic antioxidants play critical role in detoxifying ROS activities of three significant antioxidant enzymes (SOD, CAT, and POD), were measured in the two transgenic lines and WT before and after freezing and drought stresses. As can be seen in **Figure 9**, the activities of the three enzymes in the two transgenic lines were higher than in the WT under normal conditions. After 20 h of freezing stress, it is noticeable that the two transgenic lines contained significantly higher enzyme activity than did the WT. When 60-days-old tobacco plants were used for the drought stress, similar results were obtained: the activities of the three detoxifying enzyme was higher in the transgenic lines than WT (**Figure 9**), consistent with the proportion to the ROS accumulation of these lines. These data revealed that overexpression of *PebNAC1* greatly influenced the activities of the antioxidant enzymes in the transgenic plants.

### Expression Analysis of Stress-Responsive Genes under Various Treatments

**FIGURE 10:**
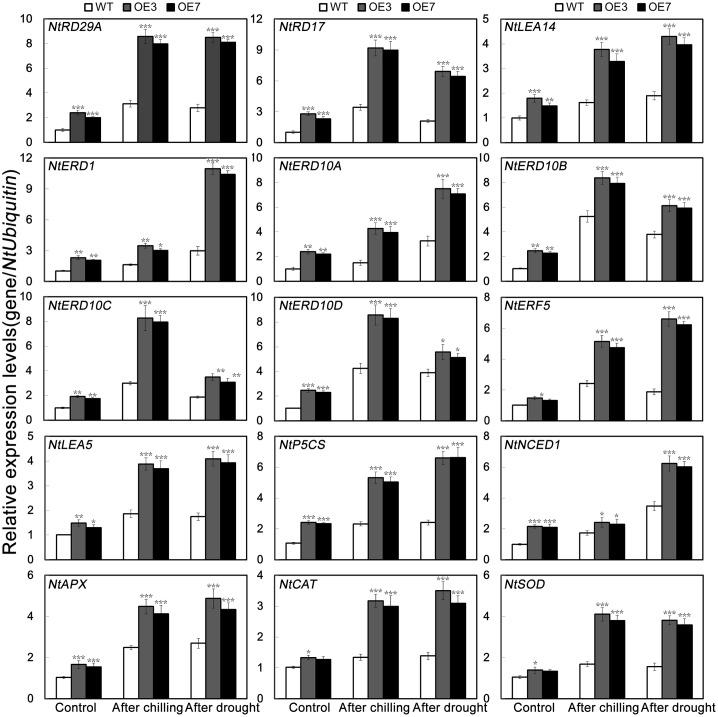
QPCR analysis of mRNA abundance of 15 stress-responsive and ROS-related genes in the transgenic lines (OE3 and OE7) and WT under normal condition and under cold and drought treatment. Asterisks indicate that the value is significantly different from that of the WT at the same time point (^∗^*P* < 0.05; ^∗∗^*P* < 0.01; ^∗∗∗^*P* < 0.001).

It has been suggested that gene expression levels are likely to be correlated with the magnitude of stress tolerance in plants (Huang et al., 2013; Li et al., 2017). To elucidate the molecular mechanism underlying the role of *PbeNAC1* in abiotic stress response, expression patterns of 15 stress responsive or ROS-related genes were compared between the WT and the transgenic lines before and after freezing stress for 20 h or drought treatment for 10 days by qPCR (**Figure 10**). These genes encode stress responsive proteins (*NtRD29A*, *NtRD17*, *NtLEA14*, *NtERD1*, *NtERD10A*, *NtERD10B*, *NtERD10C* and *NtERD10D*, *NtERF5* and *NtLEA5*), enzymes for biosynthesis of proline (*NtP5CS*) or abscisic acid (ABA; *NtNCED1*), or enzymes involved in ROS scavenging (*NtCAT*, *NtSOD*, and *NtAPX*). As shown in **Figure 10**, transcript levels of these genes were evidently higher in the transgenic lines than in the WT under normal growth conditions. When exposure to freezing or drought stress caused up-regulation of the transcript levels of the analyzed genes in all lines, but OE3 and OE7 still had a higher expression level in comparison with the WT. These results suggests that *PbeNAC1* may function in abiotic stress tolerances through regulating or interacting with the stress-related genes, although it remains unclear how such regulation or interaction can be established.

### PbeNAC1 Physically Interacts with PbeDREBs

**FIGURE 11:**
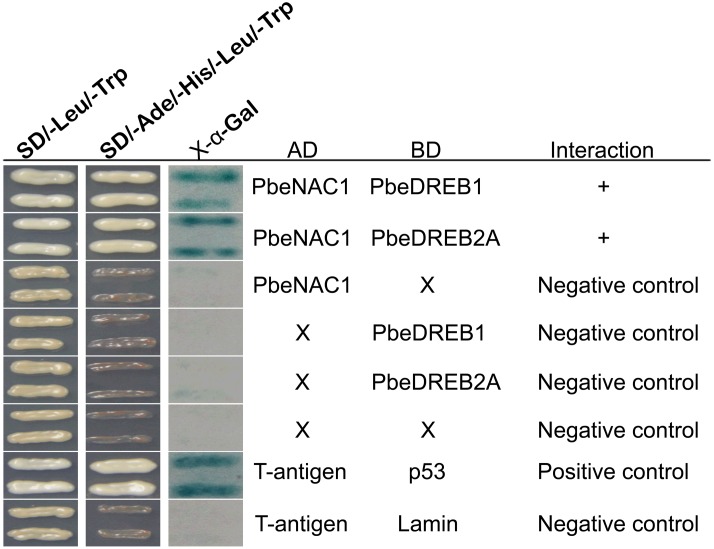
Interaction between PbeNAC1 and PbeDREB1/2A verified by yeast two-hybrid analysis. Y2H was carried out by selective growth with an X-gal activity assay. Yeast cells co-transformed with pGAD-PbeNAC1 + +pGBKT7, pGBK-PbeDREB1/2A + pGADT7, pGADT7 + pGBKT7, pGADT7-T + pGBKT7-Lam or pGADT7-T + pGBKT7-53 were used as negative or positive controls, respectively.

NAC TFs have been considered as a cofactor for transcription regulation in humans and yeast (Hu et al., 2006). To address how PbeNAC1 regulates transcription in pear, Y2H assay was carried out to identify proteins that may function in a complex with PbeNAC1. A total of 21 putative stress-responsive proteins (Supplementary Table S2) were carried out to identify whether these proteins could interact with PbeNAC1. The putative PbeNAC1-interacting proteins included TFs (ICE1, bHLH1, DREB1, ABF3, DREB2A, and MYC2), kinases (CIPK9 and MAPK6), stress-associated proteins (arginine decarboxylase, and LEA5), and others. Interestingly, two proteins (PbeDREB1 and PbeDREB2A) of them were shown that they can interact with PbeNAC1. In addition, previous work showed that DREBs plays a key role in abiotic stresses (Sakuma et al., 2006a; Champ et al., 2007; Qin et al., 2008; Wisniewski et al., 2011; Shan et al., 2014), which prompted us to focus on these two proteins. First, the interaction between two putative proteins (PbeDREB1 and PbeDREB2A) and PbeNAC1 was confirmed by Y2H assay. As shown in **Figure 11**, only yeast cells of the positive control and co-transformant with bait and prey grew normally on SD/–Leu/–Trp/–Ura medium, whereas the negative control did not survive.

**FIGURE 12:**
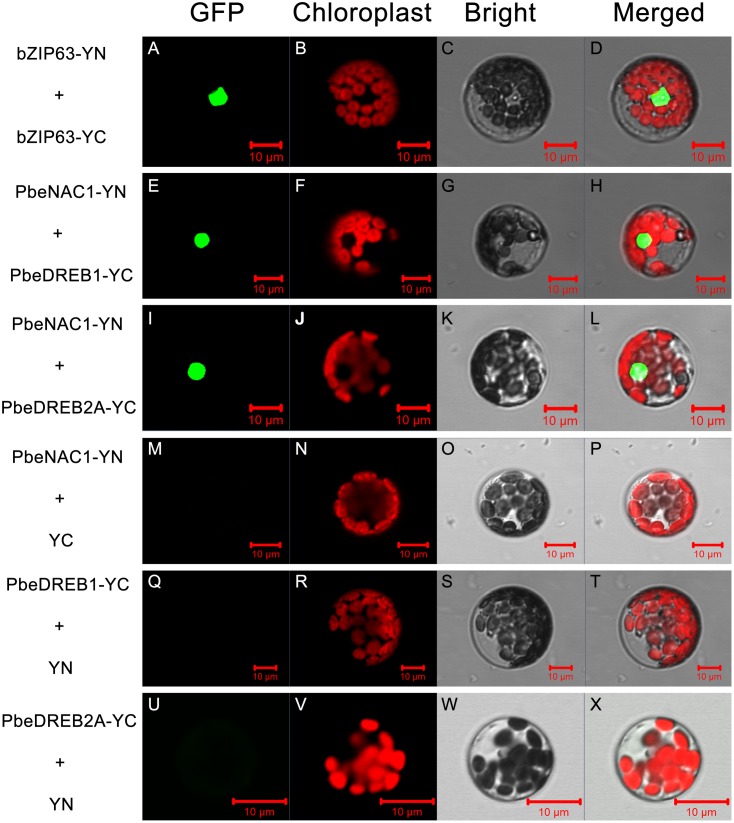
Bimolecular fluorescence complementation assay using *Arabidopsis* protoplasts. Positive **(A–L)** and negative controls **(M–X)** were bZIP63–YFP^C^+bZIP-YFP^N^ and PbeNAC1-YFP^N^+YFP^C^, respectively. All the images were taken by confocal microscopy.

A BiFC assay was further performed to verify the Y2H analysis. Green fluorescence was observed in *Arabidopsis* protoplasts, transformed with vectors containing PbeNAC1-pSPYNE and PbeDREB1-pSPYCE; PbeNAC1-pSPYNE and PbeDREB2A-pSPYCE, or with vectors of the positive control (**Figure 12**). Whereas there was no green fluorescent in the cells transformed with vectors of the negative control (PbeNAC1-pSPYNE and pSPYCE; PbeDREB1-pSPYCE and pSPYNE, or with the vectors of PbeDREB2A-pSPYCE and pSPYNE). Both the Y2H and BiFC assays indicated that PbeNAC1 could interact with PbeDREB1 or PbeDREB2A.

## Discussion

NACs, representing a large TF family specific to plants, are composed of various members in different plants. NAC proteins form an integral part of signaling web exerting crucial roles in regulating arrays of physiological or biochemical processes. However, so far, only a limited number of NACs have been characterized in a few plants, predominantly in the model plants, while functional roles and underlying mechanisms of a large number of NACs remain elusive, particularly in the perennial fruit trees. Therefore, elucidation of the functions of NACs in perennial fruit trees, such as *P. betulifolia*, will advance our understanding on the diverse roles of NACs. Here, we demonstrated that *PbeNAC1* of *P. betulifolia* plays a positive role in drought or cold tolerance by interacting with PbeDREBs and activating the expression of stress-responsive genes.

The defining feature of NACs is the presence of a highly conserved N-terminal DNA-binding domain composed of a NAC signature and a diversified C-terminal domain. Multiple sequence alignment suggests that *PbeNAC1* may be clustered into the typical NAC group because it has a highly conserved NAC domain, consisting of 125 amino acids at the N-terminus. Sequence characteristics in this region have been widely observed in typical NAC proteins from other plant species, suggesting that they may share common biological features in terms of DNA binding. However, the C-terminal sequences were extensively distinct from each other, which may account for the distinct functions of different proteins derived from various clades.

A quick induction of *PbeNAC1* mRNA levels was caused by cold, dehydration, and salt. These abiotic stresses-induced expression pattern of *PbeNAC1* was supported by the earlier studies, such as rice (Hu et al., 2006), Soybean (Hao et al., 2011), wheat (Mao et al., 2012). Banana (Shan et al., 2014) and Citrus (Wu et al., 2016). Surprisingly, we found that transcript level of *PbeNAC1* was not induced by ABA, which is contrary to previous studies (Hu et al., 2006). The reason for this may be mainly attributed to the disparity in plant species. Compared with salt stress, low temperature and dehydration caused higher induction of *PbeNAC1* transcript levels, which prompted us to do-indepth study and elucidate its roles in cold or drought tolerance.

Increasing evidence indicates that NAC proteins are involved in plant responses to abiotic stresses (Hu et al., 2006; Wu et al., 2016). Such as, the mRNA abundance of many NAC genes show substantial changes in response to various abiotic stresses and that overexpression of a single NAC gene leads to noticeable changes in abiotic stress tolerance. A number of NAC genes associated with cold or drought tolerance have been reported from diverse species, such as banana, citrus, rice and *Arabidopsis*. For example, overexpression of SNAC1/2 in rice conferred improved drought or cold tolerance and grain yield under drought conditions (Hu et al., 2006, 2008). Similarly, overexpression of MaNAC1 from banana markedly improved cold resistance in transgenic plants (Shan et al., 2014). In our work, it is demonstrated that overexpression of *PbeNAC1* in tobacco conferred enhanced cold tolerance under freezing stress, as shown by lower EL, higher survival rate, and reduce MDA content in transgenic plants overexpressing the gene, in agreement with earlier findings (Shan et al., 2014). Apart from the cold treatment used herein, overexpression of *PbeNAC1* rendered the tobacco transgenic plants better phenotypic morphology, such as leaf withering, lower EL and lower ROS levels than wild type under either dehydration or drought. Taken together, these finding corroborate earlier reports demonstrating that transformation of NAC genes could lead to enhanced tolerance to drought (Hu et al., 2006, 2008).

It is well documented that excessive accumulation of ROS causes cell oxidative damages and destroy cellular function, so plants maintain their ROS at low levels by scavenging overproduction of ROS (Miller et al., 2010; Suzuki et al., 2012). In our work, transgenic lines accumulated lower levels of ROS relative to WT after drought and cold treatments. As ROS accumulation under stressful situations largely depends on the balance between generation and concurrent scavenging, the lower level of ROS in the transgenic lines may be due to more effectively scavenging systems relative to the WT. In order to detoxify stress-induced ROS, plants have evolved a set of ROS detoxification system, in which several enzymes play essential roles, maintaining the ROS homeostasis at a favorable situation so that the cells are less influence by the oxidative stress. ROS scavenging in plants is mainly attributed to antioxidant enzymes, such as SOD, CAT, and POD (Miller et al., 2010). In this work, transgenic plants had higher activities of three antioxidant enzymes (CAT, POD, and SOD), implying that they have a very powerful antioxidant enzyme scavenging system to eliminate ROS produced compared with the WT.

To cope with unfavorable environmental constraints plants modulate the expression of a large number of stress-responsive genes, constituting an important molecular basis for the response and adaptation of plants to stresses (Umezawa et al., 2006). To gain a deeper understanding the molecular basis of enhanced stress tolerance of the *PbeNAC1*, mRNA levels of 15 stress-responsive genes were picked up to monitor by qRT-PCR before and after either freezing or drought treatment, including stress responsive proteins (*NtRD29A*, *NtRD17*, *NtLEA14*, *NtERD10A*, *NtERD10B*, *NtERD10C* and *NtERD10D*, *NtERD1*, *NtERF5* and *NtLEA5*), enzymes for biosynthesis of proline (*NtP5CS*) or ABA (*NtNCED1*), or enzymes involved in ROS scavenging (*NtCAT*, *NtSOD*, and *NtAPX*), Previous study reported most of these genes or these homologs have been responsive to abiotic stresses. QPCR analysis showed that the expression level of these genes were higher in the transgenic plants compared with those of WT under either freezing or drought stress, consistent with earlier reports in which overexpression of a transcription activator may have an ability to activate a series of target genes that functions in encounters and adaptations to abiotic stresses (Wang et al., 2011; Huang et al., 2015b; Li et al., 2017). Accumulating evidence has demonstrated that RD29A, RD17, and LEA14 proteins function in responses or adaptations to drought stress by promotion of water retaining, maintaining cell membrane integrity and preventing cell from damage (Sakuma et al., 2006a,b; Qin et al., 2008). In this work, overexpression of *PbeNAC1* in tobacco conferred enhanced dehydration and drought tolerance, which suggests that the transgenic plants might maintain membrane integrity or efficiently bind water in a better way. This assumption was supported by the assessment of higher expression of some stress-responsive genes (NtLEA5/14 and NtERD10A/B/C/D), which were assumed to be involved in counteracting cellular drought (Hundertmark and Hincha, 2008). In this study, induction of *P5CS* of the transgenic plants were stronger than that of the WT before and after either freezing or drought stress. This result suggested that the transgenic lines resulted in increased more proline, allowing them to counteract the adverse effects of these stress, in agreement with earlier findings (Silva-Ortega et al., 2008). It is interesting that the *NtNCED1* was induced to higher levels in the transgenic lines than WT after drought stress. There was no difference between WT and OE lines in terms of NCED gene expression, but NCED gene expression did increase with a low temperature treatment. In the future, extra work is need to check whether overexpression of NCED from other plants could confer enhanced tolerance to cold. It is found that the induction of these three genes (*NtAPX*, *NtSO*D, and *NtCAT*) related to ROS scavenging, were stronger in the *PbeNAC1*-overexpressing lines than in the WT before and after either freezing or drought stress. It is noticeable that the expression levels of these three genes were positively correlated with the higher activities of antioxidant enzymes (SOD, POD, and CAT) relative to WT under the freezing and drought stresses. These data suggest that the PbeNAC1 could improve drought/cold tolerance by, partially, the more efficient ROS-scavenging systems in the transgenic lines.

## Conclusion

We demonstrate here that over-expression of PbeNAC1 in tobacco result in clear increase of drought and cold tolerance, which also acts as a positive regulator of some stress-responsive genes expression from tobacco. It is known that NAC proteins can regulate gene expression by recognizing and binding to a DNA sequence with a core 4-mer motif, CACG, that allows them to regulate downstream genes (Simpson et al., 2003; Tran et al., 2004). In this case, it is possible that some of them might be activated on transcriptional level as direct target of PbeNAC1. In the future, extra work should be carried out to decipher the connection between these genes so as to gain more insight into the molecular mechanisms underlying PbeNAC1 function in stress tolerance. Furthermore, PbeNAC1 plays a key roles in modulating drought or cold tolerance at least in part, due to ROS scavenging. Totally, 21 putative PbeNAC1-interacting candidates were carried out to identify, among which two proteins (PbeDREB1 and PbeDREB2A) belonged to DREB families that were identified as two true members in the interactome. Taken together, these results indicated that *PbeNAC1* functioned in mediating cold and drought tolerance by interacting with PbeDREBs (PbeDREB1 and PbeDREB2A). Establishment of the NAC1-DREBs network provides valuable understanding of molecular basis of *NAC1* and widens our understanding of the complex cold signaling network.

The present work demonstrate that overexpression of *PbeNAC1* significantly modified drought and cold tolerance, but in the future, more work is required to experimentally unravel other components related to *PbeNAC1* so as to gain a clear-cut silhouette of the major hub in the network.

## Accession Numbers

GenBank accession numbers for the genes which were monitored transcriptional levels are as follows: *NtRD29A* (TC115477 in DFCI database), *NtRD17* (XP_016502582.1), *NtLEA14* (XP_009621630), *NtERD1* (Gu144573), *NtERD10A* (AB049335), *NtERD10B* (AB049336), *NtERD10C* (AB049337), *NtERD10D* (AB049338), *NtERF5* (AY655738), *NtLEA5* (AF053076), *NtP5CS* (HM854026), *NtNCED1* (HM068892), *NtAPX* (U15933.1), *NtCAT* (U93244.1), *NtSOD* (AB093097).

## Author Contributions

XH designed the research. XH and CJ performed the experiments. SZ, KL, XX, HC, JH, YC, YW, and HZ proofread this manuscript. XH and CJ wrote the manuscript. All authors read and approved the final manuscript.

## Conflict of Interest Statement

The authors declare that the research was conducted in the absence of any commercial or financial relationships that could be construed as a potential conflict of interest.

## Footnotes

1 http://peargenome.njau.edu.cn/

2 http://www.expasy.org/tools
