# Supplementary material for: A Novel NAC Transcription Factor, PbeNAC1, of Pyrus betulifolia Confers Cold and Drought Tolerance via Interacting with PbeDREBs and Activating the Expression of Stress-Responsive Genes
Source: Front Plant Sci. 2017 Jun 30;8:1049. doi: 10.3389/fpls.2017.01049 (PMC5491619; doi:10.3389/fpls.2017.01049)
Supplement: Supplementary file 1 [file Presentation_1.PDF]

Supporting information

Figure S1 Generation and molecular identification of the transgenic tobacco lines overexpressing *PbeNAC1*. (A) Illustrative diagram of the *PbeNAC1* overexpression fusion vector construction (pCAMBIA1301-*PbeNAC1*) used for tobacco transformation. LB, left border; HYG, hygromycin; 35S, cauliflower mosaic virus 35S promoter; RB, right border. (B) Genomic PCR characterization of *Hyg*-resistant T_0 generation tobacco plants using specific primers. (C) Overexpression analysis of PbeNAC1 in eight transgenic lines by RT-PCR. The *NtUbiquitin* gene was used as an internal control for normalizing the expression levels.

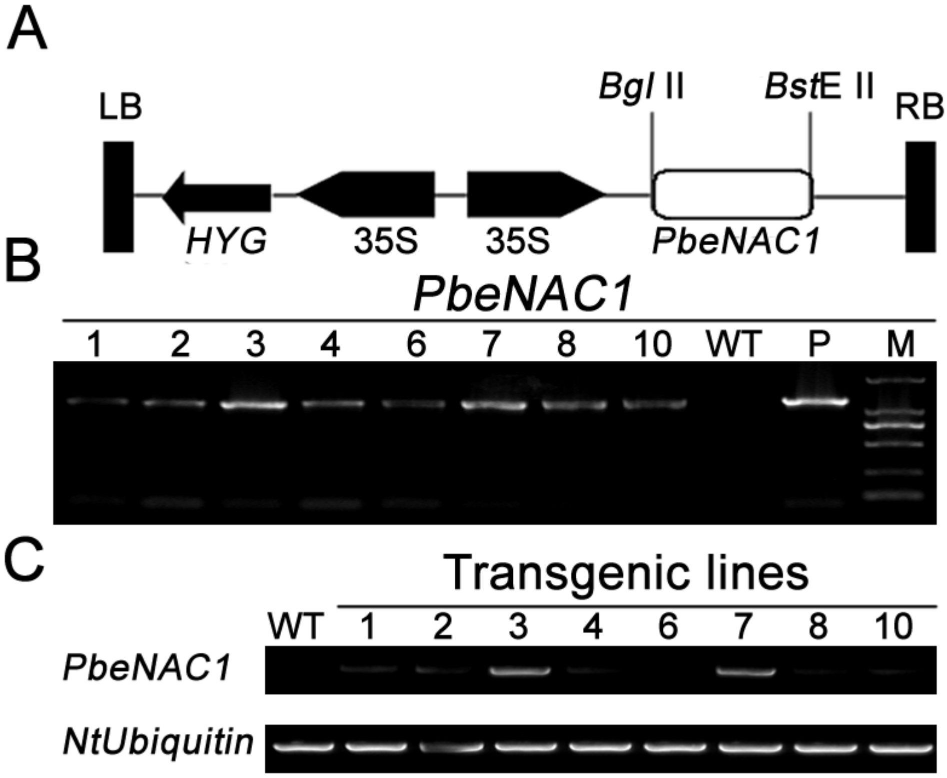

Table S1 Primer sequences used in this study.

Genes	Primers	Sequences (5′-3′)	
		Forward	Reverse
PbeNAC1	GSP1	GCACTCGGGATTAGAAAGGC	TTGATGCTGCTTGTGGTGGT
PbeNAC1	GSP2	AGATCTTCAACGATTCAGAAGAGGCA (*Bgl*II siteis underlined)	GGTACCCTCTGTGGTGCATAACTTGG (*BstE*II siteis underlined)
PbeNAC1	GSP3	CCCGGGATGAGCAGAGGAGGAGACGAGC (*Sma*I siteis underlined)	GAGCTCCAATTGGGCGTGGAGGTAGGAG (*Sac*I siteis underlined)
PbeNAC1	GSP4	AGATCTATGAGCAGAGGAGGAGACGAGC (*Bgl*II site is underlined)	ACTAGTCAATTGGGCGTGGAGGTAGGAG (*Spe*I site is underlined)
PbeNAC1	GSP5	GAATTCATGAGCAGAGGAGGAGACGAGC (*EcoR*Isite is underlined)	CTCGAGCAATTGGGCGTGGAGGTAGGAG (*Xho*I site is underlined)
PbeDREB1	GSP6	GGATCCATGGATGGTTGCTCTAATTACTAC (*BamH*Isite is underlined)	GTCGACATCGGAAAAACTCCATAAGGC (*Sal*Isite is underlined)
PbeDREB2A	GSP7	GAATTCATGGGAGCGTATGATCAAG (*EcoR*Isite is underlined)	GGATCCTCACATTTCCATCGAATAGTT (*BamH*Isite is underlined)
PbeNAC1	GSP8	TCTAGAATGAGCAGAGGAGGAGACGA （*Xba*Isite is underlined）	GGATCCCAATTGGGCGTGGAGGTAGG (*BamH*Isite is underlined)
PbeDREB2A	GSP9	TCTAGAATGGGAGCGTATGATCAAG (*Xba*Isite is underlined)	GGATCCTCACATTTCCATCGAATAGTT (*BamH*Isite is underlined)
TUB-b2		TGGGCTTTGCTCCTCTTAC	CCTTCGTGCTCATCTTACC
NtUbiquitin		AGCTACATGACGCCATTTCC	CCCTGTAAAGCAGCACCTTC
NtRD29A		AGCTGATCCGGAGAAGAGAATAAC	CATCGGTGCACCCCAATAGT
NtRD17		TGACCGTGGATGTGGATTGT	TGCTGTGAGTGCGGTGAAGT
NtLEA14		ATGTTAGATGTGCCAGTGAAAG	TCCACCCTTCCATAAATCGG
NtERD1		GTTTACCCAATGTTTCTGATGC	GCTGCCTACCTTGCTCTA
NtERD10A		TGTTCGTGAAGAAATGTCGC	GATGTTCCCCACCAAGTATG
NtERD10B		GCAATCCCATTCGTCAAACC	TGTTCCCCACCAAGTATGCC
NtERD10C		ACGTGGAGGCTACAGATCGTGGTTTG	TCTCCACTGGTACAGCCGTGTCCTCAC
NtERD10D		GAGGACACGGCTGTACCAGT	GCGCCACTTCCTCTGTCTT
NtERF5		ACACTTCATTTTCACATTCCAACTT	TCTTCTAATTCTTGACCATGGCTAC
NtP5Cs		TTCCAGACGTCTTCAGGCAC	TCATATCCGGCCTGTTGAGC
NLEA5		CTCTAACTCCAAACTCATCTCTGC	CAAAACCCCAGATTCAAGAC
NtNCED1		AAGAATGGCTCCGCAAGTTA	GCCTAGCAATTCCAGAGTGG
NtCAT		AGGTACCGCTCATTCACACC	AAGCAAGCTTTTGACCCAGA
NtSOD		GGTGTTTCCAGTGGCGGACG	TCCTCCCCTCAGCTACGGGGTAT
NtAPX		CAAATGTAAGAGGAAACTCAGAGGA	AGCAACAACTCCAGCTAATTGATAG

Table S2 Information about the PbeNAC1-interacting proteins.

AD	BD	Interaction
PbeNAC1	PbeICE1	-
PbeNAC1	PbebHLH1	-
PbeNAC1	PbeProDH1	-
PbeNAC1	PbeESK1	-
PbeNAC1	PbeDREB1	+
PbeNAC1	PbeCIPK9	-
PbeNAC1	PbeCBL1	-
PbeNAC1	PbeADC	-
PbeNAC1	PbeHHP1	-
PbeNAC1	PbeLEA5	-
PbeNAC1	PbeABF3	-
PbeNAC1	PbeDREB2A	+
PbeNAC1	PbeABI2	-
PbeNAC1	PbeMYC2	-
PbeNAC1	PbeMAPK6	-
PbeNAC1	PbeMYB2	-
PbeNAC1	PbeMYB4	-
PbeNAC1	PbeERF3	-
PbeNAC1	PbeERF5	-
PbeNAC1	PbeWRKY11	-
PbeNAC1	PbeWRKY21	-
